# Measuring alignment of structural proteins in engineered tissue constructs using polarized Raman spectroscopy

**DOI:** 10.1371/journal.pone.0324704

**Published:** 2025-05-30

**Authors:** Maedeh Lotfi, Hui Zhou, Janny Piñeiro Llanes, Ghatu Subhash, Chelsey S. Simmons, Malisa Sarntinoranont

**Affiliations:** 1 Department of Mechanical and Aerospace Engineering, Herbert Wertheim College of Engineering, University of Florida, Gainesville, Florida, United States of America; 2 J. Crayton Pruitt Family Department of Biomedical Engineering, Herbert Wertheim College of Engineering, University of Florida, Gainesville, Florida, United States of America; Advanced Materials Technology Research Institute, National Research Centre, EGYPT

## Abstract

Measures of structural protein alignment within biological and engineered tissues are needed for improved understanding of their mechanical behavior and functionality. We advance our method of measuring protein alignment using polarized Raman spectroscopy (PRS). It provides a promising alternative to conventional microscopy-based methods as it is non-destructive and allows analysis of extracellular components without additional protein labeling. Previously, we used a machine learning-based alignment metric to compare the extent of alignment between various soft tissues. This study demonstrates that PRS can be successfully used to provide a sensitive measure of alignment in engineered tissues despite the challenges of water-dominated spectra, which have limited prior efforts. A framework for capturing spatial variation of the amplitude and angle of bulk protein alignment was developed. Engineered tissue constructs were generated using collagen type-I solutions seeded with mouse myoblast (C2C12) cells. Tissue alignment was introduced as samples contracted over 12 days of culture. PRS measures of alignment within three selected regions captured a 32% change in extent of alignment and a 30° change in angle between center and corner regions. A computational model was used to bridge between discrete fiber measures of alignment determined with standard immunofluorescence microscopy and our PRS technique. The model applied contraction strains within a hyperelastic continuum to model cell contraction, and model-derived alignment measures showed good agreement between microscopy and PRS measures. Overall, our study provides additional analysis tools for quantifying alignment with PRS and showed the high potential of this PRS technique to non-invasively measure spatial variation within engineered tissues. Such measurement tools are needed to engineer regional alignments aimed at capturing specific mechanical and functional capabilities.

## Introduction

Tissue engineering holds the promise of replacing damaged organs or missing tissues. However, the field faces a number of challenges that impede implementation, including manufacture of native hierarchical structures and matching mechanical and failure properties [1]. To overcome these barriers, a better understanding of the relationship between microscopic structure and macroscopic mechanical behavior is needed. New structural characterization tools would aid in successful design, implementation and assessment of novel biomaterials with complex structure [2,3].

A key structural component in many load-bearing soft tissues is collagen, which plays a dominant role in determining tissue mechanics and function [4]. Collagen’s hierarchical organization—ranging from molecules to fibrils to fibers—is tailored for specific functional needs in tissues such as tendon, cornea, and skin. These tissues exhibit distinct collagen alignments depending on their physiological roles [4]. For example, the parallel alignment of collagen fibers in tendon enhances longitudinal strength [5], the random layered organization in the skin maximizes compliance, while the precise layered organization in the cornea facilitates both strength and transparency [6]. In structures with more complex loading patterns, alignment also varies with position within the tissue, e.g., lamellae in annulus. In engineered tissues, the goal is often to recapitulate or engineer collagen structures to mimic such structural organization. Consequently, tools that allow quantification of direction and extent of tissue alignment are useful [5]. Previous studies have shown that alignment changes can be induced by cell-mediated contraction, which alters fiber organization over time. Several groups have quantified these effects using imaging techniques such as fluorescence microscopy, particularly under varying levels of contraction [2,7–21]. For instance, O’Rourke et al. quantified fiber alignment at different locations in tissue using fluorescence images for different levels of contraction [2,18].

Various imaging techniques have been employed to characterize structural alignment of protein structures in tissues and engineered tissue constructs. Light-based imaging methods suffer from limited penetration depths, e.g., 100–300 µm for second harmonic generation [22]. Polarized light microscopy is specifically suited for examining bundle fibers in collagen-rich tissues [23,24]. Techniques such as fluorescence microscopy and Förster resonance energy transfer also require the introduction of fluorescent labels. Scanning electron microscopy (SEM) while providing high resolution data, lacks the ability to study samples in their native physiological environment and provides only limited information about internal structure. Fourier transform infrared spectroscopy (FTIR) has also been employed for alignment studies, however its incompatibility with water presents a limitation [25,26]. Raman spectroscopy (RS) is a non-invasive approach that has been developed to characterize the bond composition of molecules. By harnessing the inelastic scattering of light, RS identifies molecular vibrational modes. This method provides advantages such as label-free preparation, high spatial resolution (less than 1 µm), high molecular specificity and hydrated testing which is ideal for measurement of cell-laden scaffolds under normal physiological conditions. The introduction of a polarized filter further allows us to determine directional dependence of molecular vibrations at each measured location, providing a basis for measuring alignment of fibers since they exhibit a highly organized hierarchical structure. At the macroscopic level, individual collagen fibers are composed of bundles of collagen fibrils, which are assembled from collections of collagen molecules which in turn consist of three peptide chains that aggregate both laterally and longitudinally. Using polarized Raman spectroscopy (PRS), we utilize the arrangement of these peptide chains to determine the alignment of fibers. Thus, a PRS-based method provides an attractive means of non-contact, real-time alignment measurements. Corresponding Raman spectra that are collected also allow for more detailed examination of extracellular matrix (ECM) component distributions [27].

To date, this PRS technique has been mainly used by our group and others to characterize the extent of matrix organization in native soft tissues [28–32]. In our previous studies, a loading coefficient was developed by employing a machine learning algorithm, specifically principal component analysis (PCA), and utilizing highly aligned muscle tissue spectra as a normalized reference for comparing alignment in different tissues. By using this loading function, the specific influence of the C = O bond, which is indicative of protein alignment, can be captured while other irrelevant chemical bonds are eliminated from consideration. We have used this combination of PRS with data science methods to determine collective alignment within native tissues, such as dermis and hypodermis [28,29]. It is less clear if a master loading function derived from aligned tissue can be used to characterize engineered tissue constructs. For example, while Bergholt et al. successfully used PRS to characterize collagen orientation in articular cartilage, they were unable to measure alignment in engineered cartilage tissues due to a dominant water signal using a similar PRS method [31].

In this study, our PRS-based method is further developed for engineered tissue constructs with high collagen concentration (4 mg/ml), known structural alignments, and PCA that included weighting for different chemical bonds over a range of wavelengths (1400−1800 cm^-1^). Controlled tissue engineering conditions are ideal for testing, and myoblast cells seeded in collagen matrices were used to generate contracted tissues with well characterized alignment. We provide improved assessment of spatial variation in alignment, by providing a framework for establishing alignment angles and introducing computational models that relate PRS measures to discrete fiber measures of alignment using standard immunofluorescence microscopy. This computational mechanics model provides a more complete spatial map of alignment. Also, models can be used as a promising tool to relate the dependency between structural alignment and mechanical characteristics such as stress and stiffness. By integrating spectral, imaging, and modeling data, we introduce a robust, label-free platform for non-destructive, real-time monitoring of collagen organization in engineered constructs. This approach offers valuable insight into structural changes over time and supports quality control during tissue fabrication.

## Methods

Myoblast cells cultured in collagen matrices generated contractile forces that deformed and induced tissue alignment in dog bone-shaped engineered tissue constructs. First, each sample underwent PRS testing, then each sample was labeled, and fiber images were captured in the same regions using immunofluorescence microscopy. Further analysis and computational modeling quantified directions and extent of alignment. Detailed methods are provided below.

### Engineered tissue construct

#### Cell culture.

C2C12 myoblasts which have the ability to mature into functional muscle cells were chosen to offer a relevant model for studying *in vivo*-like cell behavior. An undifferentiated mouse myogenic C2C12 cell line (ATCC, No. CRL-1772) at passage 5–10 was maintained in basal Dubelcco’s modified Eagle’s medium (DMEM, Gibco Life Technologies) supplemented with 10% v/v fetal bovine serum (FBS, Gibco Life Technologies) and 1% v/v penicillin-streptomycin (Pen/Strep, HyClone SV30010) at 37°C under 5% CO_2_. The growth medium was replaced every 2 days, and the C2C12 myoblasts were passaged upon reaching 70% confluency.

#### Preparation and functionalizing PDMS mold constructs.

**Fig 1:**
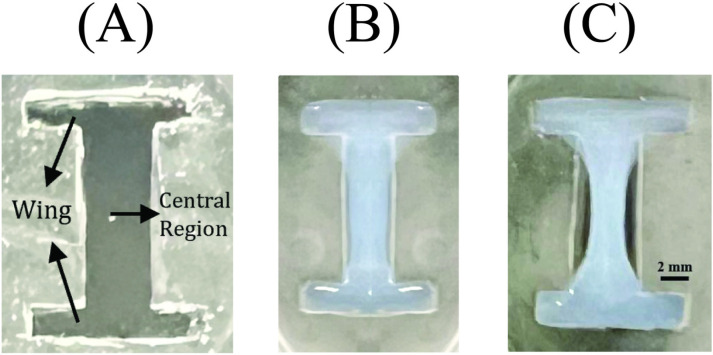
Engineered tissue construct. (A) Fabricated PDMS dog-bone shaped mold; (B) Precursor collagen was poured into adhesion wings (2 mm × 10 mm), then precursor collagen mixed with C2C12 cells was poured into the central region (4 mm × 10 mm). Differentiation media was added. (C) Deformed engineered tissue construct after 2 days of incubation.

Once seeded into dog bone-shaped tissue molds, myoblasts generate contractile forces that result in sculpted tissues. These engineered tissues were fabricated as described in Mondrinos et al. [7] with certain geometrical modifications. Briefly, 1-mm thick polymer molds were fabricated using polydimethylsiloxane (PDMS) (Sylgard 184, Dow Corning) at a weight ratio of 10:1. A degassed PDMS mixture was poured into a Petri dish and cured at 64°C for 4 hrs. Two rectangular regions (2 mm × 10 mm) were cut out to create anchorage regions spaced at a distance of 10 mm apart. Selectively functionalizing the surface of the two anchorage wings provided heterobifunctional cross-links that increase the adhesion strength between the hydrogel and the PDMS mold. The PDMS mold was washed, sterilized within a biosafety hood, and dried before adding a Sulfo-SANPAH solution (1mg/mL, Thermo Scientific) to each wing. The mold and solution were immediately exposed to light using a cure system (ELC-500 UV Cure Unit, UVitron company) set to 15 mW/cm^2^ (50% lamp intensity) for 300 seconds. The system was configured to emit light at a wavelength of 485 nm to initiate photoreaction under controlled intensity conditions. The sulfo-SANPAH solution was then aspirated, and this process was repeated four times to increase adhesion. PDMS adhesion wings were rinsed with 1X phosphate buffered saline [PBS, 155.17 mM NaCl (9000 mg/L), 1.54 mM KH₂PO₄ (210 mg/L), 2.71 mM Na₂HPO₄-7H₂O (726 mg/L) with pH adjusted to 7.4] three times, dried, and placed on a Parafilm substrate to prevent the adhesion of collagen solution to the tissue culture plastic. PDMS from the central region (4 mm × 10 mm) was cut out between the two functionalized adhesion wings to generate the final dog-bone test shape. Within the dog-bone mold, PDMS adhesion wings provided fixed boundaries which supported the tension stress generated after the addition of collagen hydrogel and cells (7). The central region was not fixed and free to deform, see Fig 1.

#### Cell seeding and engineered tissue fabrication.

Tissues precursor solution was prepared by mixing a high-concentration of rat tail collagen type-I (Corning) with 0.2% acetic acid (Glacial, Fisher Scientific), Matrigel (Corning), 10X DMEM (Sigma), HEPES (Gibco) solution, and PBS. Physiological pH and osmolarity were controlled using sodium bicarbonate (Fisher Bioreagents). The precursor solution contained a 4 mg/ml final concentration of collagen I and 10% v/v of Matrigel. C2C12 myoblasts were gently admixed to the precursor solution at a density of $5\times 10^{5}$ cells/ml. In parallel, to obtain a precursor solution with equivalent concentration but without cells, the same protocol was followed with the addition of PBS as a substitute for the additional volume of cells. The precursor solution without cells was loaded to the top and bottom anchorage chambers and allowed to start the thermogel process for 5 min at 37°C. Then, precursor solution containing cells was loaded into the central region. Samples were incubated for 1 hr. at 37°C for gelation. The process was timed to ensure that construct seeding occurred within 5 min of completing surface functionalization. After gelation, cultures were immersed in a standard cell culture medium and incubated for 24 hrs. at 37°C and 5% CO_2_. After 24 hrs, underlying Parafilm was peeled away and the media was replaced with a differentiation medium composed of DMEM supplemented with 2% v/v horse serum (Gibco, 26-050-088), 1% v/v Psen/Strep, and 0.01% v/v insulin-transferrin-selenium (ITS, Gibco 41-400-045). The addition of ITS to myoblast has been previously shown to increase the differentiation rate by preventing glucose-mediated inhibition of myogenesis [33]. The differentiation media was partially changed every 3 days for a total of 12 days in cell culture. Several Engineered tissue samples incubated under identical conditions underwent fluorescence imaging and PRS testing.

#### Immunofluorescence staining protocol.

For immunofluorescence staining of actin filaments, samples were kept attached to the PDMS mold constructs to minimize damage and track selected locations. All engineered tissue samples were rinsed 3 times with 1X PBS and fixed with 4% paraformaldehyde (PFA) overnight at 4 °C. Next, samples were rinsed with PBS, permeabilized with 0.1% Triton X-100 (Sigma Aldrich), and blocked with 5% bovine serum albumin (BSA) overnight at 4 °C. To visualize myotube formation, samples were incubated with MF20 primary antibody (DSHB, 1:200) diluted in 5% BSA. The following day, the samples were rinsed three times with 1X PBS and incubated with the secondary antibody (ab150113, Thermo Scientific, 1:200), and phalloidin for 3 hrs. to ensure antibodies penetrated into the tissues. Finally, samples were rinsed 3 times with 1X PBS, carefully detached from the PDMS construct using surgical tools, and mounted on thin coverslips to allow visualization of the cytoskeletal and myotube alignment. Tissue samples were imaged using a Keyence microscope (Keyence BZ-X810; Keyence Corporation, Itasca, IL, USA) through a 40X objective (NA = 0.6, S Plan Fluor ELWD ADM 40XC, Nikon, EI Segundo, CA, USA) with a 400X total magnification and an image resolution of 0.1887 µm per pixel. Z-stacks were acquired at a step size of 1 µm. Multiple images were collected, and one representative central slice was selected from each sample for imaging analysis.

#### Tissue alignment from immunofluorescent images.

**Fig 2:**
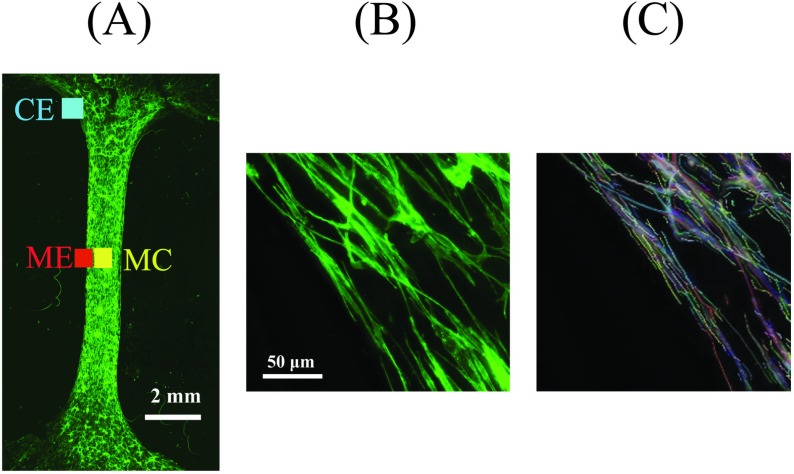
Immunofluorescence labeled engineered tissue. (A) Three selected locations, at the corner edge (CE), middle edge (ME), and middle center (MC) were chosen to measure protein fiber alignment. (B) Immunofluoresence (IF) image of labeled fibers in 100μm×100μm ROI at the corner edge at day 12 of incubation. (C) Corresponding CT-FIRE segmented fibers shown as colored lines overlaid on a gray scale IF image.

The fiber tracking and extraction (FIRE) method developed by Wu et al. [34,35] and Stein et al. [36] has been previously used to extract fiber-level information from images of matrices. This method enables analysis of critical fiber-level parameters including fiber lengths, numbers, and curvatures to make orientation histograms and calculate alignment indices [34–36]. Adding appropriate pre- and post-image processing significantly improves image fiber extraction [37]. Actin fiber directions and numbers were calculated from immunofluorescence images using the open-source software CT-FIRE (V3.0 Beta, UW-Madison) which uses a curvelet-denoising filter followed by FIRE (see Fig 2). Compiled with custom MATLAB subroutines that read in image files, the CT-FIRE program quantified fiber angles, length, straightness, and widths. Measurements were applied on 100 µm × 100 µm regions of interest (ROI) at select locations at the corner edge (CE), middle edge (ME), and middle center (MC) within the test sample. The number and direction of fibers within each ROI were used to make orientation histograms.

We calculated an alignment index (S) to provide a scalar measure of alignment for the ROI population of discrete fibers with varying directions (38) by defining,


$$S=2\left\langle \cos^{2}\theta \right\rangle -1$$ (1)

where the θ is the angle between each fiber and the mean fiber axis, which serves as a reference direction. Mean fiber axis is calculated by finding the major axis of an ellipse which is fitted to all the fibers (39). This axis is calculated easily if all the fibers distribute symmetry around a random axis [38]. The $\left\langle \cos^{2}\theta \right\rangle$ in Eq. 1 denotes the quantity averaged over all fibers given by


$$\left\langle \cos^{2}\theta \right\rangle =\frac{\sum N(\theta )\cos^{2}\theta }{\sum N(\theta )}$$ (2)

where N(θ) is the number of fibers oriented at angle θ. For a distribution of randomly- and perfectly- aligned fibers, S will vary between 0 and 1, respectively [38–40]. Together, S and *θ* were used to compare variation of alignment magnitude and the angle of alignment, respectively, at different locations in labeled images of tissue constructs. Lastly, standard deviation (SD) variation was calculated for distributed fiber angles in each ROI as a standard method to evaluate the degree of alignment and misalignment of fibers. SD provides a measure of how much the fiber angles deviate from the mean angle with each ROI.

### Tissue alignment from polarized Raman spectroscopy (PRS)

PRS was performed using a Renishaw InVia spectrometer with a 532 nm argon-ion laser at 50 mW power capacity, with approximately 5 mW reaching the sample surface (10% of output). The incident laser was initially polarized horizontally. A linear polarizer was placed behind the Rayleigh filter in the scattered beam to select the parallel scattered light component with respect to the polarization of incident light. Spectra were collected using an 1800 lines/mm VIS grating, providing high spectral resolution across the range of interest. Single crystal silicon was used to calibrate the spectrometer for Raman shifts (peak position at 520.5 cm^−1^). Linearity of the Raman calibration was measured with an internal neon calibration source showing precision and accuracy of Raman wavenumbers better than 0.4 cm^−1^ after correcting for off-axis and chromatic aberration. Spectra were acquired using a charge-coupled device camera (Andor EMCCD) and collected using WIRE®4.4 workstation software (Renishaw plc, England) in extensive modes ranging from 1400 to 1800 cm^-1^.

**Fig 3:**
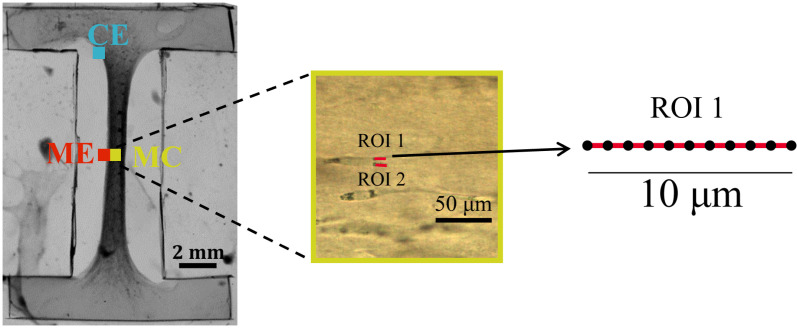
Selected ROIs. Two ROIs (ROI 1 and ROI 2) were selected within the middle center (MC) location. For each ROI, PRS was measured at 11 points equally spaced along a 10-micron line. 12 replicates were obtained at each point (measurement repeated 12 times). 264 spectra were collected at 22 different points in MC.

Within each sample, PRS measurements were taken in three different select locations (CE, ME, and MC). Within each location, two ROI were selected (ROI 1 and ROI 2). For each ROI, PRS was measured at 11 points equally spaced along a 10 µm-line. 12 replicates were obtained at each point (measurement repeated 12 times). 264 spectra were collected at 22 different points in each CE, ME, and MC location, see Fig 3. PRS measurements were performed on fixed samples (4% paraformaldehyde) submerged in PBS to further preserve hydration at the measurement surface and at room temperature, neglecting thermal effects. A 20X water-immersion objective was for use in PBS. With the linear polarizer in place, each location within each sample was measured at different polarization angles between the laser polarization direction and the sample alignment axis. A 10 s acquisition time was used for each measurement. Samples were rotated between 0° to 180° in 30° increments (see Fig 1 in [29]). Rotated measurements were used to capture the angle of alignment. Rotation was restricted to 150° based on the natural periodicity of protein fiber distributions. To confirm accurate angle placement at each rotation step, a mechanical stage with an angle-marked ruler was used. Additionally, reference images of each region of interest (ROI) were taken at 0° and visually compared after each rotation. These images were also digitally rotated and overlaid to quantitatively assess angular differences. This process confirmed angular accuracy within ±5°, and only measurements with consistent morphology and position were accepted. After collecting spectra of the tissue constructs, PBS background was subtracted in WIRE®4.4 workstation software using an eleventh-order polynomial fit. Aluminum foil was used as a tissue sample sublayer to remove the underlying layer effect on tissue spectra. In MATLAB, a Savitzky-Golay finite impulse response (FIR) smoothing filter with a third-order polynomial and a frame length of 11 was used to smooth the spectra. A standard normal variate (SNV) analysis was performed to normalize the spectra. Afterwards, PCA was applied to reduce the dimensionality of complex Raman spectra into a new set of orthogonal variables (principal components) that capture the most variance in the data.

**Fig 4:**
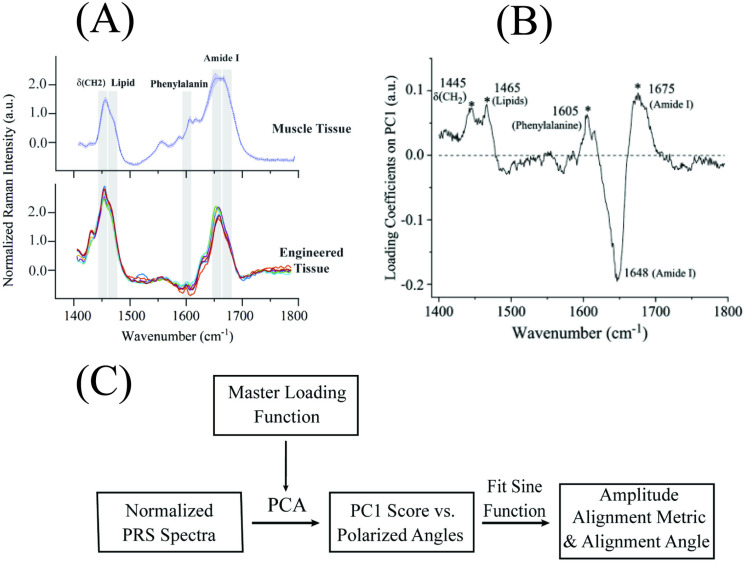
Polarized Raman spectroscopy. (A) Raman Spectra of native muscle tissue, and engineered tissue construct in the 1400−1800 cm^-1^ range. Both samples showed similar Raman peaks indicating same chemical bands such as CH2 bending band, CH2/CH3 deformation, C = C stretching, C = O stretching that were assigned to lipids and carbohydrates, collagen and lipids, amino acids (e.g., tryptophan, phenyl- alanine, etc.) and proteins, respectively. (B) Principal component loading curve (master loading function) for primary principal component (PC1) was derived based on highly aligned muscle layer spectra (110 spectra total). Bands in the loading curve correspond to CH2, lipids, phenylalanine, and Amide I vibration modes, respectively, which are known to be present in both native soft tissue and engineered tissue construct (29). (C) Flow diagram of steps used to determine protein fiber alignment via polarized Raman spectroscopy (PCA = principal component analysis).

In soft tissues, collagen comprises different chemical bonds with wavelengths in the range 1400–1800 cm^-1^, including CH2, lipids, phenylalanine, and amide I bands correspond to 1445, 1465, 1605, 1630–1656 cm^−1^ and 1665–1685 cm^−1^, respectively, see Fig 4. The C = O bond within peptide chains is directed at 90° to the collagen fibrils which are aligned with the collagen fibers. Our PRS methodology determines orientation of protein chains using differences in Raman band intensities under varying polarization angles based on significant increases in peak intensity at certain angles due to alignment of C = O bonds. We were able to infer the alignment of protein fibers within native tissue and tissue constructs [41–45]. As engineered tissue constructs contain varying matrix components, PRS coupled with multivariate PCA was used to decompose the Raman spectrum into a single variable. PCA was applied to provide the primary principal component (PC1) for a tissue construct using a loading coefficient that was based on the reference sample for every normalized intensity at a given wavenumber (see Fig 4) [46]. The percentage of variance explained by each principal component was calculated separately for the corner edge, middle edge, and middle center regions. PC1 consistently accounted for the majority of variance in all regions, followed by decreasing contributions from PC2 and PC3. Cumulative variance plots were used to justify the use of PC1 alone as the dominant descriptor of alignment-related spectral variation.

Highly aligned muscle tissue spectra were used as the normalized reference for comparing tissue alignment. Spectral range was limited to 1400–1800 cm^-1^ since the loading coefficient was calculated in the same range. The glass substrate background has a strong contribution in the wavelength 700–1200 cm^−1^, hence wavelengths from 600–1300 cm^−1^ were cut from analysis of the loading coefficient to avoid conflation of the signal with the glass background. Spectra in wavelengths from 1400–1800 cm^−1^ can be easily subtracted from glass substrates without compromising spectral features of cells and proteins [29]. Overall, PCA converted the normalized spectra of different polarized angles into a single score (PC1) as a function of polarized angle that can be quantified and related to the degree of matrix alignment. The score for each spectrum represents its position along the principal component axes, effectively reducing the dimensionality of the data while preserving the most significant variance. As in our previous studies [28–30], alignment in a region was estimated by fitting a sine function to this output,


$$PC1={PC1}_{0}+A\sin\left(\frac{\pi (\varphi -\varphi_{c})}{90\circ }\right)$$ (3)

where *PC1*_*0*_ is a *PC1* offset for the sine function. The amplitude of the sine fit, *A,* is the amplitude alignment metric which provides a measure of extent of alignment. φ is the Raman polarization angle (in degrees) and $\varphi_{c}$ is the phase shift. The polarization angle corresponding to the maximum principal score value, i.e., maximum *PC1*, was used to provide a PRS-based alignment angle, *β*_PRS_, that describes the dominant direction of protein fibers (${\beta_{PRS}=\varphi }_{c}+45\circ$). Together, *A* and $\beta_{PRS}$ quantify PRS alignment magnitude and direction at different spatial locations in the tissue constructs. The alignment magnitude is based on the PRS spectra only.

Statistical significance was determined in JMP statistical software (Pro 16.1.0, SAS, NC), using a one-way ANOVA followed by Tukey-Kramer’s post-hoc test to determine the difference of alignment between different regions in the same tissue construct. To quantify the uncertainty in the alignment metrics, 95% confidence intervals (CIs) were calculated for each tissue region. The CI for each region was computed using $CI=mean\pm t_{crit}.\frac{SD}{\sqrt{n}}$, where the mean represents the average alignment metric (A), SD is the standard deviation across measurements, n is the number of spectra collected each region (n = 22), and $t_{crit}$ is the critical value from the Student’s t-distribution. For a two-tailed test at a significance level of α = 0.05 and 21 degrees of freedom (n – 1), the critical t-value was 2.080. This approach provides a 95% confidence range around the mean, reflecting the interval in which the true population value is expected to lie. Confidence intervals were used to report precision in alignment parameters. In order to compare with fluorescence imaging and computational modeling alignment metrics, we also introduced an alignment aspect ratio $,\gamma =\frac{A}{A_{\max}},$ where *A*_*max*_ is the maximum *A* measured inside the sample.

### Model of cell-induced tissue deformation and alignment

**Fig 5:**
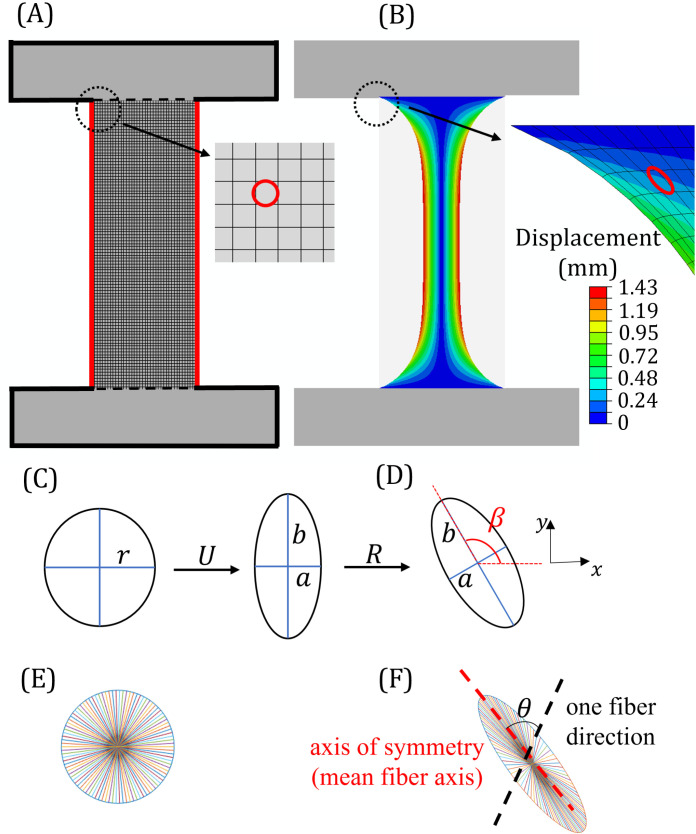
Computational model. In the computational model, alignment of engineered tissue was determined based on predicted deformation of an embedded circle (r = 0.05 mm) at a material point. (A) FE model used to predict deformation of sample when a shrinkage strain was applied. Red lines = free boundaries; black lines = fixed boundaries. (B) Predicted shape and magnitude of displacement contour ($\varepsilon_{s}=0.6,E=700Paand\upsilon =0.4)$ after 12 days incubation; (C) Process to calculate stretch (*U*) and rotation (*R*) of each circle with embedded “fibers.” *a* and *b* are the minor and major ellipse axes. (E-F) Equivalent alignment index, *S*, was determined by introducing discrete, embedded “fibers” to each material point circle; (E) before and (F) after deformation. The predicted redistribution of embedded fibers was used to calculate and *S* and θ, the angle between each fiber and the axis of symmetry (*θ* = 0).


Cell-induced deformation of the engineered tissue (Fig 1) was modeled computationally to match measured tissue deformations and predict bulk changes in tissue alignment at different spatial locations. A plane stress finite element (FE) model was developed using the commercial software package Abaqus (Dassault System Simulia Corp., 2019) and tested to show mesh independence (100 µm × 100 µm elements). A plane stress condition was considered since thickness of samples was approximately 0.1 times the sample length. The geometry and boundary conditions matched those of the engineered tissue sample before incubation, see Fig 5A. Collagen fibers, which are the major structural component of engineered tissue constructs, were considered to be at high enough concentration to act as a continuum phase. To capture deformation due to cell contraction, the cell-laden region of the engineered tissue was subjected to a linear shrinkage strain $\varepsilon_{s}$. To simulate tissue shrinkage in Abaqus, thermal loading (coupled temperature-displacement) was utilized where a thermal strain was applied to generate equivalent shrinkage strain ($\varepsilon_{s}$). Thermal strain ($\varepsilon_{s}=\varepsilon_{t}$) was assigned a constant value that resulted in different levels of tissue shrinkage. No other temperature-dependent relations were applied. Engineered tissue was modeled as a compressible, hyperelastic, and neo-Hookean material defined by strain energy (ψ) as,

$$\psi =\frac{E}{4(1+\nu )}\left(\overset{―}{I_{1}}-3\right)+\frac{E}{6(1-2\nu )}{\left(J^{el}-1\right)}^{2}$$ (4)

The elastic volume ratio ($J^{el}=\frac{J}{J^{s}}$) relates the total volume ratio (J) and the shrinkage volume ratio ($J^{s}={\left(1+\varepsilon_{s}\right)}^{3}$). ${\overset{―}{I}}_{1}$ is the first strain invariant. Mechanical properties, elastic modulus (E=700Pa) and Poisson’s ratio (ν=0.4) were assigned based on measured values for collagen tissue [47]. FE models simulated deformation of samples for increasing values of $\varepsilon_{s}$ up to 0.6 (in increments of 0.1 or smaller). Best fit $\varepsilon_{s}$ at day 12 incubation was determined by minimizing the difference between predicted areas of tissue shrinkage and measured areas from sample images, see Fig 5B.

Once the deformed tissue shape was predicted (after fitting to shrinkage), a geometrical treatment was used to understand local deformation in the neighborhood of a point [48] and predict fiber alignment. Collagen fibers were assumed to be perfectly embedded and to align with the continuum phase. Before deformation, material points in the model domain were mapped to uniform, isotropic circles. After deformation, circles became deformed quadratic ellipses [48] whose shape and orientation angle were used to predict amplitude and angles of embedded protein fiber alignment. The stretch tensor ($U=\sqrt{F^{T}F}$where F is the deformation gradient) was used to calculate the major and minor axes of the ellipse, *a* and *b*. We introduced a local alignment factor, $\alpha =\frac{b}{a}$, that varied between 1≤α<∞ for istotropic and perfectly aligned tissues, respectively. The rotation tensor ($R=FU^{-1}$) was used to calculate the major axis angle (principal axis) of each ellipse, representing the angle of fiber alignment, *β*_model_ (see Fig 5C–D). Together, *α* and *β*_model_ were used to quantify alignment magnitude and direction of embedded collagen fibers predicted by the computational model, respectively. The local alignment factor was only based on tissue deformation predictions. To compare with PRS metrics, we also introduced an alignment aspect ratio $,\gamma =\frac{\alpha }{\alpha_{\max}},$ where *α*_max_ is the maximum α predicted inside the sample.

Additional analysis used custom MATLAB subroutines to compare model predictions with discrete immunofluorescent imaging measures of *S* (Eq. 1). At each material point (each circle before deformation), we introduced a distribution of “fibers” that were perfectly embedded and had the same mechanical properties as the surrounding continuum, see Fig 5E–F. The number of fibers was set equal to the estimated number of fibers extracted from immunofluorescence images (*N* = 150) and were distributed uniformly in a radial circular pattern. After deformation into an ellipsoid, angles between fibers and the major axis of symmetry of the fibers were estimated using the same analysis as described in above to determine equivalent measures of S and *θ*.

## Results

### Tissue alignment from immunofluorescent images

**Fig 6:**
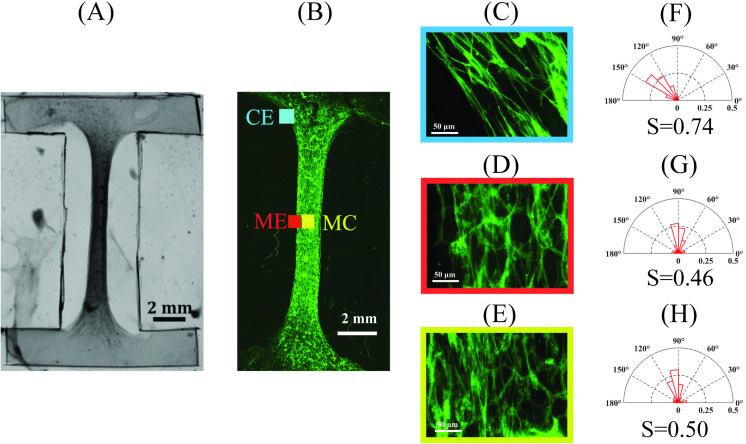
Tissue alignment from immunofluorescent images. (A) Engineered tissue construct after 12 days incubation (4 mg/ml collagen I, 5 × 10^5^ cells/ml C2C12 myoblast cells, 5-10 passages) exhibiting cell-induced deformation and fiber alignment. (B) Immunofluorescent image of engineered tissue construct, showing F-actin in cytoskeleton fibers stained with phalloidin; Enlargement of (C) corner edge, (D) middle edge, and (E) middle center regions; (F-H) Polar histograms and calculated S determined from CT-FIRE at (F) corner edge (θ = 129.1° ± 25°), (G) middle edge (θ = 89.2° ± 38°), and (H) middle center (*θ* = 96.8° ± 37.8°) locations (100 µm × 100 µm regions).

Engineered tissue samples exhibited cell-induced shrinkage with corresponding changes in shape and tissue alignment, see Fig 6A–B. After 12 days incubation, area shrinkage in the central region was measured to be ~ 60%. Polar histograms show fiber density and orientation of labeled F-actin fibers within three select locations: corner edge, middle edge, and middle center regions (~150 fibers in 100 µm × 100 µm fields of view), see Fig 6C–H. Corresponding alignment indices for these locations were calculated to be *S*=0.74 (CE), 0.46 (ME), and 0.50 (MC). Within select regions, mean and standard deviation of the angle of distributed fibers in each ROI were determined to be *θ = *129.1° ± 25° (CE), *θ = *89.2° ± 38° (ME), and *θ = *96.8° ± 37.8° (MC). Mainly, the corner edge region showed a distinctly higher extent of alignment of the protein fibers, mostly aligned between *θ* = 120–150°. In middle regions, fibers were found to vertically align (θ~90∘).

### Tissue alignment from PRS

Raman spectra for the engineered tissue showed spectra peaks that were closely aligned with that of the reference muscle tissue (see Fig 4). Alignment of maximum peaks underscores the ability of engineered tissue to replicate major structural protein characteristics found in native tissues. The percentage of total variance explained by each principal component was calculated separately for each region. PC1 consistently accounted for the largest proportion of variance across all three tissue regions, supporting its role as the dominant descriptor of alignment-related spectral features. Specifically, PC1 explained 74.3% of the total variance in the corner edge, 60.6% in the middle edge, and 62.0% in the middle center. These results indicate that the main alignment signal is strongly captured by the first component. Over 80% of the total spectral variance is captured within the first three components in all regions, while PC2 and PC3 accounted for an additional 7% to 23% of the variance. PC2 and PC3 were not systematically associated with alignment-related spectral features, based on spectral loadings and polarization angle trends. Therefore, PC1 as it captured the largest proportion of variance, was used for all further PRS analysis of alignment.

**Fig 7:**
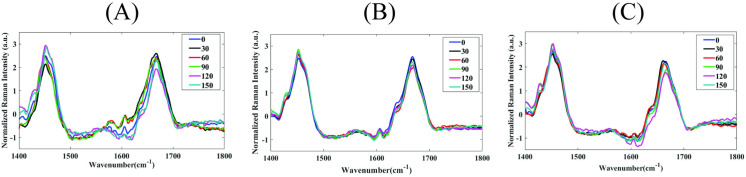
Raman Spectra. Mean of normalized Raman spectra for different laser polarization angles (legend values are in degrees) over the 1400−1800 cm^-1^ range at (A) corner edge, (B) middle edge, and (C) middle center locations (see Fig 6) in the engineered tissue construct (based on 264 total collected spectra at each location of the same tissue sample, spectra also collected for two different samples).

**Fig 8:**
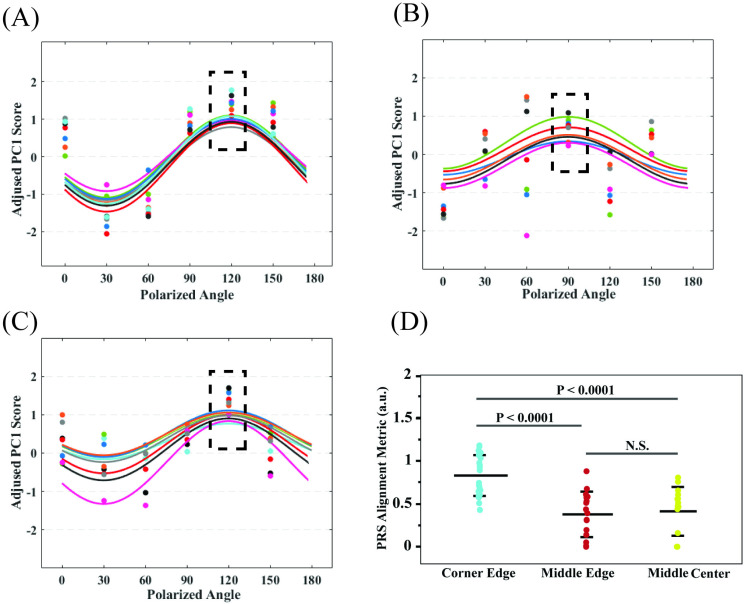
Tissue alignment from PRS measurements. Variation of PC1 with polarized angle was fit to sine functions at select locations (a subset of the full 22 points per region is shown in A–C for clarity): (A) corner edge, (B) middle edge, and (C) middle center regions. Matching colors indicate the same measurement location for different polarized angles used to fit each sine curve in A-C. (D) The fitted amplitude is the alignment metric which was found to be significantly higher at the corner edge. PRS was conducted on fixed tissues that were fixed 12 days after the start of incubation. Error bars indicate one standard deviation of the mean. P-value determined by one-way ANOVA followed by Tukey-Kramer’s post-hoc test. Lower p-value revealed a distinguished alignment between the different locations.

Within select regions, collected PRS (Fig 7) was processed to determine PC1 scores at each polarized angle, see Fig 8A–C. Sine wave fits provided measures of the amplitude alignment metric within similar regions: *A* = 0.83 ± 0.05 (95% CI: 0.81–0.85) for CE, 0.37 ± 0.06 (95% CI: 0.34–0.40) for ME, and 0.40 ± 0.07 (95% CI: 0.37–0.43) for MC. The corresponding alignment aspect ratio were: γ=1(CE), 0.43(ME), and 0.48(MC). Metrics for 22 points at each select location were compared in Fig 8D and show PRS was able to determine a greater extent of alignment at the corner edge compared to middle regions (p-value <0.0001), which is consistent with results from fluorescent imaging of labeled protein fibers. Phase shifts were found to be $\varphi_{c}$ = 75° (CE), 45° (ME), and 75° (MC), and corresponding angles of alignment were found to be $\beta_{PRS}$= 120° ± 15°(CE), 90° ± 15° (ME), and 120° ± 15° (MC).

### Tissue alignment from computational modeling

**Fig 9:**
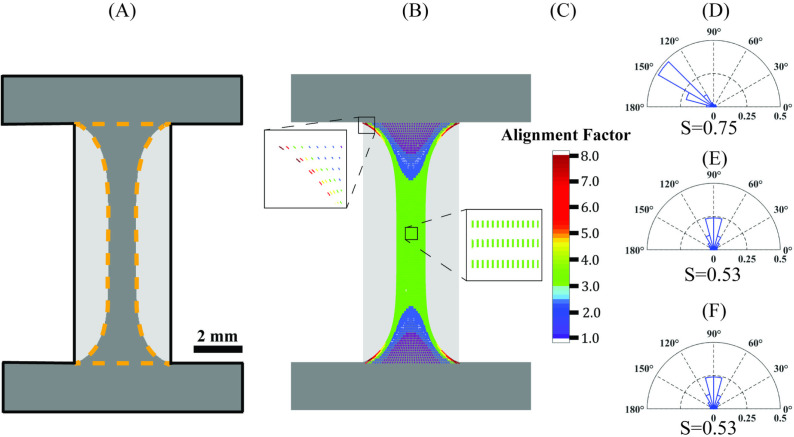
Tissue alignment from the computational model. (A) Deformed central region was fit to match experimentally measured area shrinkage after 12 days incubation. (B) Contour map of alignment factor (α) based on the calculated deformation gradient. Contour values of α are averaged over 100 µm × 100 µm elements. Expanded boxes show the predicted alignment angle of embedded fibers ($\beta_{model}$) scaled by α at CE and MC. Equivalent polar histogram and alignment index (S) at (C) corner edge, (D) middle edge, and (E) middle center calculated from modeled distributed fibers (averaged 150 fibers for a 100 µm × 100 µm element size, same as experiment).

Fig 9 shows simulated deformation of the construct due to uniform cell contraction (12 days after incubation) within cell-laden regions. $\varepsilon_{s}=0.6$ was found to be the best fit shrinkage strain by matching area changes in the cell-laden region of the sample (60% area shrinkage). Contour maps of the predicted strain field and the corresponding alignment factor, α, are shown in Fig 9B–C. Based on averaging local measures of ellipse deformation at each point, the extent of alignment for similar regions was determined: α=7.8(CE) and α=3.5 (MC & ME). The related aspect ratio for these regions was found: γ=1 (CE) and γ=0.44 (MC & ME). Maximum extent of alignment is predicted in corner regions. The angle of alignment based on the principal direction of each deformed ellipse represents the direction of the most significant number of fibers at each location. Within select regions, angles of alignment were determined to be $\beta_{model}=145\circ$ (CE) and $\beta_{model}=90\circ$ (MC & ME), see Fig 9B.

By introducing fibers at each point in the model, we calculated equivalent S values. Fig 9D–F shows polar histograms for these fiber distributions after model deformation: S=0.75 (CE), S=0.53 (MC & ME). Polar histograms based on introduced discrete fibers for these selected locations show *θ* = 120° to 150° (CE) and *θ* ≅ 90° (MC & ME). Calculated computational alignment indices matched with measures from labeled fluorescence images in the same regions, showing higher alignment in the corner edge compared to middle regions. Also, the determined alignment angles were comparable between PRS and labeled fluorescence measures in these same regions.

### Measurement comparison

**Fig 10:**
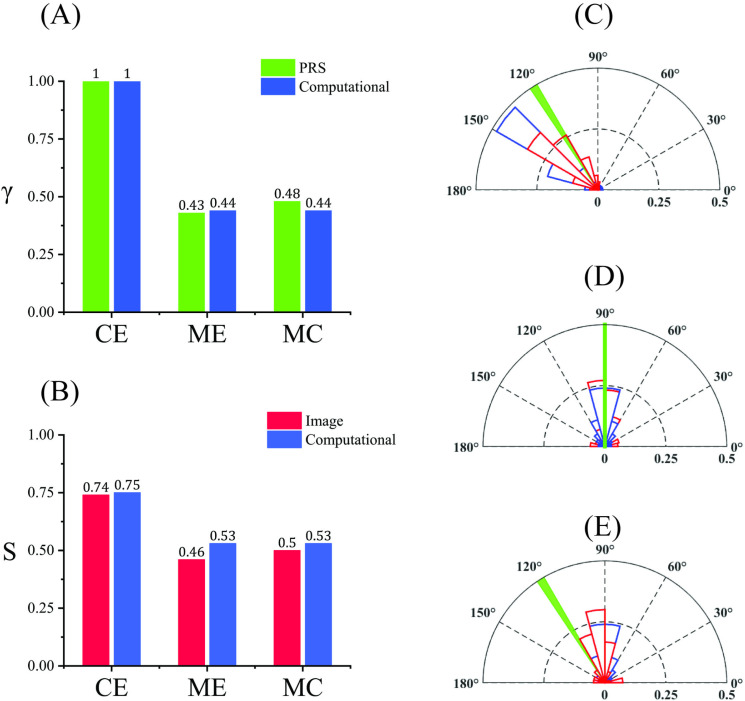
Comparison of protein alignment metrics. (A) γ and (B) S for selected locations using PRS, fluorescent imaging and computational methods. Comparison of alignment angles at (C) corner edge, (D) middle edge, and (E) middle center. Red = image alignment angles, blue = computational alignment angles, green = PRS alignment angles.

Normalized, PRS alignment aspect ratio along middle regions ($\gamma_{PRS}=\frac{A}{Acorner}=0.45and0.48$) were similar to fluorescently measured and computationally predicted ratios of alignment metric ($\gamma_{model}=\frac{\alpha }{\alpha_{corner}}=0.44and0.44$), showing the ability of PRS to capture a similar spatial variation, see Fig 10A. We also used PRS to determine a dominant direction of fiber alignment (based on C = O orientation in protein fiber molecules and calculated phase shift of PRS). Measured PRS alignment angles at corner region ($\beta_{PRS}=120\circ$), middle edge ($\beta_{PRS}=90\circ$) and middle center ($\beta_{PRS}=120\circ$) matched those of reference fluorescent images at the same locations. Fibers are distributed between 120° to 150° (129.1° ± 25°) at the corner edge, around 90° (89.2° ± 38°) at the middle edge, and between 90° to 120° (96.8° ± 37.8°) at the middle center in reference labeled images, see Fig 10C–E. Small differences in the angle of alignment at some locations is related to the big increment angle (±30°) used in PRS measurements.

## Discussion

Our study leverages the advanced data evaluation method of principal component analysis to better understand complexities of protein tissue alignment in Raman spectra. This methodology distinguishes our approach, and we were able to use PCA of Raman spectra to identify alignment-related peaks without the need for prior assumptions about peak locations. This approach is especially useful for heterogeneous biological samples where traditional deconvolution methods introduce greater uncertainty, particularly when examining tissue constructs with varying protein content. Moreover, the ability to derive alignment information without a priori peak specification makes the methodology adaptable and robust by avoiding reliance on single PRS peaks. We have used the combination of PRS and a PCA machine learning algorithm to determine spatial variation of alignment in engineered tissues building upon our prior feasibility studies that show PRS can detect alignment differences between isotropic (S~0.19) and highly aligned collagen in engineered tissue constructs (S~0.96) [28]. This method relies on the use of a master loading function based on highly aligned native muscle tissue with well characterized alignment in one primary direction. Since the engineered tissues were designed to develop heterogeneous alignment through cell-mediated contraction over time, no regions of maximal alignment were assumed a priori. For this reason, using the loading function from the engineered tissues could introduce uncertainty or bias, especially in regions with low or unknown alignment levels. Also, the Raman spectra for the reference tissue and engineered tissues had the same highest spectral peak in the range 1400–1800 cm^-1^ as they comprise the same chemical bonds, including CH2, lipids, phenylalanine, and amide I bands correspond to 1445, 1465, 1605, 1630–1656 cm^−1^ and 1665–1685 cm^−1^, respectively. Confirming the same biochemical composition between engineered tissue and native muscle tissue allowed us to apply the previously derived loading coefficient in PCA to compute PC1 for engineered tissue constructs. This direct spectral comparison not only validated the use of the master loading function, but also reinforced the ability of PRS to detect meaningful alignment signals in engineered tissue environments.

A major strength of this work is its ability to overcome a common limitation in the field—namely, the dominance of water-related signals in engineered tissues, which has previously hindered the successful application of PRS. Earlier studies, such as Bergholt et al. [31], reported difficulty measuring alignment in engineered cartilage due to overwhelming water signals. In contrast, our study demonstrates that, through appropriate tissue design (e.g., higher collagen concentration), fixation, and PCA-based spectral analysis, PRS can reliably quantify alignment in engineered tissues even under hydrated conditions. We believe this PRS method can be used to capture alignment variation in most engineered tissues since amide I and CH2 bands used by the master loading function is common in all soft tissues. To test this, future studies should test samples with lower collagen content.

Developed methods provide a framework for using PRS to generate spatial maps of protein alignment within specimens. The PRS method was able to distinguish differences in relative alignment in contracted tissue constructs between aligned central regions (*A* = 0.40 ± 0.07 (MC) and *A* = 0.37 ± 0.06 (ME)) and more aligned corner regions (*A* = 0.83 ± 0.05) similar to fluorescence image analysis of these regions. Additional metrics were used to quantify alignment at these select locations. Previous studies using PRS mainly verified proteins alignment qualitatively through fluorescence imaging, employing metrics like the amplitude alignment metric or principal PC1 to indicate regions of higher or lower alignment [29,31]. These approaches identify samples that are more aligned but lacked a quantitative relationship between PRS data and fluorescence images. To provide comparative results, it was important to use similar measurement location. Alignment index (S) quantified fiber alignment over a 100 µm × 100 µm ROI in order to calculate the average distribution of fibers in each select location. The measurement area was consistently used in analysis of immunofluorescent images and computational model results. To make alignment measures more comparable, this ROI was based on PRS measurements (22 points were randomly chosen in 100 µm × 100 µm ROI). The PRS alignment metric (*A*) was calculated based on continuous characteristics (intensity of spectra), so this metric was averaged for these 22 points (spectra for each location was averaged for 12 accumulations). Given the different nature of alignment measures, a computational model was needed to relate S and A measures. While not essential for PRS validation purposes, the FE model provides improved assessment of spatial variation in alignment, by providing a quantitative framework to relate PRS measures to immunofluorescence microscopy measures of discrete fiber angles. An alignment aspect ratio (γ) was introduced as a comparable parameter between PRS alignment metric (*A*) and computational alignment factor (α).

In Fig 10, we present a comparison of alignment metrics measured by PRS and those measured from immunofluorescence images. While there is some discrepancy between measured protein fiber alignment angles obtained from PRS and those derived from the immunofluorescence images, computational model analysis is able to demonstrate that the derived fiber angles show a level of agreement when processed using the same computational framework. There are several factors that contribute to PRS measurement error that we plan to focus on in future studies to better show accuracy. Mainly, the limited number of angles tested (increment size = 30°) results in a lower angular resolution with Raman spectroscopy. Future studies will focus on increasing the number of angles to improve angular resolution. The disparity between depth-averaged PRS results and depth-specific imaging results can also introduce differences. PRS effectively averages alignment data over the full thickness of the sample. In contrast, the imaging techniques used in our study captures protein alignment data at specific depths and tissue thickness (1 micron thickness in central regions of the sample). We expect relatively uniform alignment through the thickness of our sample; however, extent of alignment may vary through thickness. Specifically, the PRS data may smooth out depth-dependent variations, while the imaging data may highlight localized alignment features that are not representative of the entire tissue thickness. In addition, inherent differences in the resolution and sensitivity of the two techniques contribute to measured differences. Raman spectroscopy, being a molecular technique, accounts for alignment at this level of through molecular bonds while fluorescence imaging was applied to actin fibers at a micron length scale. In addition to differences in scale, fibers measured by PRS, and fluorescent imaging may be for different populations of proteins. This highlights the compositional aspects of tissue and underscores the challenges and benefits of a multi-modal framework.

Computational mechanics models are useful tools for predicting tissue alignment and provide a more complete spatial field. In these constructs, tissue deformations and corresponding changes in alignment were introduced by cells as they attached and contracted. A similar mechanics-based modeling approach has been used previously by Mondrinos et al. to predict deformation of engineered tissue constructs over time [7]. They used a contraction of spring model to capture tissue shrinkage and focused on determining changes in tissue stiffness, rather than changes in alignment. As collagen comprises over 80% of the tissue, we considered fibers to be perfectly embedded into ECM in the model and realignment of these fibers to match directions of maximum deformation of the bulk tissue (continuum directions of maximum stretch). Many different computational models have been developed based on continuum, fiber mixtures, and discrete fibers to study the role of protein fiber alignment on deformation behavior [2,7–21]. Other modeling studies that simulate networks of discrete protein fibers have shown fiber angles can deviate from the deformation of the bulk of ECM tissues [17,49]. Such non-affine deformation likely introduces angle variations smaller than what we are currently able to measure with PRS. The continuum model used here mainly captures bulk engagement of the majority of collagen fibers. Such continuum models are computationally less intensive and include the entirety of the test specimen.

Computational predictions of bulk tissue alignment and angle of alignment were found to compare well with reference measures within selected regions. Indeed, the computational model provided a bridge for quantitively comparing PRS results and image analysis data. By introducing isotropic ‘fibers’ at each location, we predicted alignment index at the corner edge (S=0.75), middle edge (S=0.53), and middle center (S=0.53) to match the same metrics from stained collagen images (measured to be 0.74, 0.46 and 0.50, respectively), see Fig 10B. In the computational model, the calculated alignment aspect ratios along middle regions ($\gamma_{model}=\frac{\alpha }{\alpha_{corner}}=0.44and0.44$ for ME and MC) were validated by comparing to aspect ratios determined by PRS at the same locations ($\gamma_{PRS}=\frac{A}{Acorner}=0.45and0.48$ for ME and MC), see Fig 10A. Angles of fiber alignment were also comparable between fluorescent imaging studies and PRS measures. In the computational model, the angles of fiber alignment were distributed between $\beta_{model}=120\circ to150$° at the corner edge, ~ 90° at middle regions, and matched labeled fluorescent images and PRS measured values, see Fig 10C–E. Alignment angles in labeled images and computational model simulations were determined based on discrete fiber angles. In contrast, alignment angles from PRS were based on a continuous measure. Therefore, applying smaller angle intervals (less than 30°), i.e., higher frequency, should result in a better match between PRS and discrete measures of alignment angle.

This PRS-based method provides a non-invasive, in-line method for assessing the spatial variation of the main protein component of extracellular matrix (ECM). It provides a new tool that may help guide efforts to recreate the native tissue complexity in engineered tissues and ensure sufficient mechanical integrity in tissue constructs. In addition, to mechanical contributions, collagen fibrils and fiber bundles interact with cells via several receptor families. Thus, changes in distribution and organization of collagen may influence cell adhesion, proliferation, migration, and differentiation [50]. PRS-based tools may also help in better understanding of these interactions. The ability to monitor fiber architecture label-free and in hydrated conditions is particularly valuable for quality control in tissue manufacturing, where destructive testing is not feasible. To further develop this method, future studies will focus on introducing higher resolution spatial mapping measures. This may be obtained with more automated specimen handling that provides improved translation and rotation of the test specimen in the PRS test system. The PRS method also needs to test over different incubation times to see if it can capture changes in protein and cellular composition with tissue remodeling. Within the computational model, we apply uniform cell density that contributes to uniform contraction. Future studies may incorporate image-based changes in cellular density that account for spatiotemporal changes in cell populations. Raman spectra can also be used to monitor changes in protein composition, and these measures may be used to account for the effect of changing composition. Future studies may also validate spatiotemporal changes in mechanical stress and properties associated with changes in fiber alignment to provide a more complete picture of mechanical properties.

## Conclusion

This study presents a framework for using PRS to interrogate spatial variation of alignment within engineered tissue constructs. We detail advanced data analysis methods and introduce alignment metrics that compare PRS to reference measures obtained from immunofluorescence protein fiber images. The developed computational model provided a more complete map of alignment in the whole tissue specimen, rather than just at selected locations. PRS was able to show significant difference in extent of alignment between select locations (7.5% difference in *A*). Capture of angles of alignment was partially successful in regions with large angle changes. Improved sample rotation methods and smaller increment angles need to be introduced to improve resolution of this measure. Based on these results, PRS is a promising, non-contact tool for capturing in-line changes in tissue alignment over the course of tissue incubation. Such tools are needed to better understand factors influencing regional fiber alignment in engineered tissues aimed at capturing specific mechanical and functional capabilities.
